# Evolution of Master of Public Health Core Curriculum: Trends and Insights

**DOI:** 10.1177/00333549241296787

**Published:** 2024-11-19

**Authors:** Cara L. Pennel, Denny Fe G. Agana-Norman, Leslie A. Stalnaker, Dana Wiltz-Beckham, Marisol Luna

**Affiliations:** 1 Department of Population Health and Health Disparities, School of Public and Population Health, University of Texas Medical Branch, Galveston, TX, USA; 2 Office of Academic Affairs, School of Public and Population Health, University of Texas Medical Branch, Galveston, TX, USA; 3 Department of Epidemiology, University of Texas Medical Branch, Galveston, TX, USA; 4 Working Partner, Houston, TX, USA

**Keywords:** academic public health, curriculum development, MPH core curriculum, master of public health

## Abstract

**Objectives:**

Revised accreditation criteria from the Council on Education for Public Health (CEPH) in 2016 prompted schools and programs of public health to shift their master of public health (MPH) core curricula. Our objective was to provide data on revisions to MPH core curricula at CEPH-accredited schools and programs of public health and other descriptive statistics on the MPH core curriculum and required courses as of 2023.

**Methods:**

We analyzed data from 67 accredited schools of public health and 130 accredited public health programs to assess changes from 2016 to 2023 in the MPH core curriculum. We examined the number of courses, the proportion of credit hours in the core curriculum, core curriculum composition, and course types.

**Results:**

Almost half (49.2%) of schools, but only 16.9% of programs, made extensive changes to their MPH core curricula, an overall increase of 153.6% from 2020 to 2023. Approximately one-fifth of schools and programs made few to no changes to their core curricula and retained core courses in the 5 former core disciplines. On average, core curriculum credit hours comprised 37.8% of total credit hours for schools and 51.7% for programs. Half (50.0%) of all programs in the sample offered single-concentration MPH degrees. Schools and programs were more likely to continue requiring traditional biostatistics (81.7%) and epidemiology (81.2%) courses in the core curriculum compared with environmental health (69.0%), social and behavioral health (61.9%), and health policy and management (42.1%).

**Conclusions:**

Most schools and programs modified their MPH core curricula, reflecting a departure from traditional public health courses toward innovative approaches to ensure knowledge and skill proficiency of graduates. Future research will determine if these curricular changes improve the knowledge and skill proficiency of public health graduates and the workforce.

Since 2016, public health education for professional degrees, including the master of public health (MPH) and doctor of public health (DrPH), has changed substantially at schools and programs of public health. While evolving public health trends, workforce needs, and public health employment opportunities may have influenced these educational changes, revisions to the accreditation requirements by the Council on Education for Public Health (CEPH) is considered a primary impetus.^1-3^

MPH degrees require at least 42 credit hours and typically comprise core courses, concentration courses, an applied practice experience, and an integrative learning experience. Prior to 2016, the CEPH accreditation criteria required that the core MPH classes reflect 5 public health core areas (biostatistics, environmental health, epidemiology, health policy and management, and social and behavioral sciences), which were often delivered through siloed didactic instruction.^4^ In 2016, academic public health shifted to a competency-based framework.^3^ This change allowed for innovation and integration in the core courses while addressing competencies in the following domains: evidence-based approaches to public health, public health and health care systems, planning and management to promote health, policy in public health, leadership, communication, interprofessional practice, and systems thinking.^3^ These domains encompass 22 foundational competencies that every accredited MPH must teach and assess as well as 12 foundational public health knowledge learning objectives.^3^ These changes have not been without some criticism, which largely relates to the absence of environmental health and, to a lesser extent, ethics-specific competencies.^5-7^

According to a 2020 MPH trends report on MPH curriculum design, 18 schools of public health (30.0%) and 3 public health programs (2.4%) had redesigned their MPH curricula with an integrated core, in which all or most of the 22 MPH foundational competencies and 12 foundational learning objectives were taught and assessed.^8^ Combined, this cohort accounted for 11.4% of the 184 accredited schools and programs of public health. That percentage has likely grown since 2020.

In addition, the 2020 MPH trends report indicated a median of 7 courses in the core curriculum.^8^ While there are no accreditation requirements for the number of courses or credit hours in the MPH core, the Framing the Future MPH report recommended that no more than one-third of the MPH degree be composed of core courses.^2^ For a 42-credit–hour MPH degree, that would be 14 credit hours.

The objective of this study was to provide data on revisions to MPH core curricula at CEPH-accredited schools and programs of public health and other descriptive statistics on the MPH core curriculum and courses as of 2023.

## Methods

We identified all CEPH-accredited schools of public health and public health programs on the CEPH website.^9^ We used web-based searches to gather the following information for each school and program:

- Total credit hours in the MPH degree,
- MPH core curriculum credit hours,
- Number of core curriculum courses,
- Composition of the core curriculum, and
- Types of courses.

For public health programs, we also documented whether the program had a single concentration. Because we could not distinguish between core courses and required courses, specifically for single-concentration MPH degrees, we recorded all required courses as core courses. The team collected MPH-related data from April through July 2023. When web-based searches did not yield all information necessary, searches of self-study reports filled gaps. We included only self-study reports using the 2016 or 2021 CEPH accreditation criteria. We included schools and programs only if all information was available on credit hours (total and core) and composition of core curriculum. We converted curricula reported in quarter hours into semester hours. Our sample included 67 schools of public health and 130 public health programs. At the time of this study, this accounted for all (100.0%) accredited schools and 81.3% of all accredited programs.^10^

We recorded all information in Microsoft Excel (Microsoft Corporation) and analyzed the following descriptive statistics:

- Mean, median, and range of the total number of core curriculum courses for public health schools, public health programs, and combined and
- Mean, median, and range of the core curriculum, by semester credit hours, in proportion to the total number of credit hours required for the degree for public health schools, public health programs, and combined.

Using the framework developed and used by CEPH in the 2020 MPH trends report, we classified the composition of the core curriculum for each school and program as follows:

- Category A: Discrete courses in the 5 former core areas, and some with an introduction to public health, with no other courses required for core (little to no change);
- Category B: Three to 5 discrete courses in former core areas, plus at least 1 additional course in or outside a former core area (some change); and
- Category C: Two or fewer discrete courses in former core areas, with disciplines outside former core areas and/or courses combined and fused with multiple former core areas (extensive change).^8^

We calculated the percentage of schools, programs, and the combined total that revised their core curriculum for each of these 3 categories. We reported percentages in aggregate by curriculum design category and compared these percentages with pre-2016 accreditation revisions and the 2020 CEPH report.

Finally, we examined the types of courses in the core curricula to understand changes in the traditional public health core discipline courses. We mapped courses to CEPH foundational competency domains based on course titles. We categorized methods courses that best aligned with the evidence-based approaches to public health domain (competencies 1-4) into the following groups: traditional biostatistics, traditional epidemiology, epidemiology and biostatistics combined, quantitative and qualitative methods (or unspecified methods courses), and stand-alone qualitative methods. The public health and health care systems domain (competencies 5 and 6) and policy in public health domain (competencies 12-15) included traditional health policy and management; health services, systems, administration, and/or management; policy and/or advocacy; and health equity or social justice courses. The planning and management to promote health domain (competencies 7-11) included traditional social and behavioral health courses and planning courses, frequently with assessment, implementation, and/or evaluation. The leadership (competencies 16 and 17) and communication (competencies 18-20) domains encompassed leadership and communications course categories. We excluded interprofessional practice and systems thinking courses from the course categories because of small numbers. Ethics and environmental health were each a course category but were not connected to a specific foundational competency domain.

The University of Texas Medical Branch Institutional Review Board (IRB) reviewed the project and determined the submission did not meet the definition of human subjects research, as defined by the regulations at 45 CFR 46.102. Therefore, the project did not require IRB approval or oversight.

## Results

The total number of courses in the MPH core ranged from 3 to 15 courses, with a mean of 7 courses and a median of 6 courses. Schools had fewer core curriculum courses (range, 3 to 12 courses) than public health programs did (range, 5 to 15 courses) (Table 1).

**Table 1.** Courses in master of public health core curriculum at accredited schools and programs of public health, 2023^a^

Measure	Schools of public health (n = 67)	Public health programs (n = 130)	Total (N = 197)
Range	3-12	5-15	3-15
Mean	6.0	7.6	7.0
Median	6.0	7.0	6.0

a All values are numbers.

The proportion of the total number of credits required for the MPH devoted to the core curriculum, in semester credit hours, ranged from 20.0% to 86.7%. The range, mean, and median were less overall for schools than for programs. The mean proportion of required core courses was 38.3% for schools and 50.6% for programs (Table 2).

**Table 2.** Proportion of master of public health core to total curriculum, in semester credit hours, at accredited schools and programs of public health, 2023^a^

Measure	Schools of public health (n = 67)	Public health programs (n = 130)	Total (N = 197)
Range	20.0-66.7	26.1-86.7	20.0-86.7
Mean	38.3	50.6	46.2
Median	35.7	50.0	42.9

a All values are percentages.

Almost half (49.2%) of all schools made extensive changes to their MPH core curricula (category C), while only 20.9% retained the 5 traditional core courses (category A). Similarly, only 19.2% of all programs retained the traditional core courses (category A); however, only 16.9% made extensive changes to their core curricula (category C) (Table 3). Sixty-five (50.0%) public health programs in the sample had single-concentration programs. Single-concentration programs were more likely than programs with multiple concentrations to maintain the 5 core discipline courses while also requiring a breadth of other courses. For example, single-concentration programs may have required a traditional social and behavioral health course and health communication, program planning, and program evaluation courses; traditional biostatistics and epidemiology courses and a research methods course; or a health policy course and a leadership and management course. From 2020 to 2023, the percentage of schools and programs making extensive changes increased by 153.6%, from 11.0% to 27.9% (Table 4).

**Table 3.** Schools and programs of public health by extent of master of public health core curriculum redesign, by category, 2023^a^

Curriculum design	Schools of public health (n = 67)	Public health programs (n = 130)	Total (N = 197)
Category A^b^	20.9	19.2	19.8
Category B^c^	29.9	63.9	52.3
Category C^d^	49.2	16.9	27.9

a All values are percentages.

b Discrete courses in 5 former core areas, introduction to public health in some cases, no other courses required for core (little to no change).

c Three to 5 discrete courses in former core areas, plus at least 1 additional course in or outside the former core area (some change).

d Two or fewer discrete courses in former core areas, disciplines outside former core areas, and/or courses combine and fuse multiple former core areas (extensive change).

**Table 4.** Comparisons of school and program master of public health core curriculum redesign before 2016 and in 2020 and 2023^a^

Curriculum design	Before 2016^b^	2020 (n = 184)	2023 (n = 197)
Category A^c^	100.0	10.0	19.8
Category B^d^	0	79.0	52.3
Category C^e^	0	11.0	27.9

a All values are percentages.

b The total number of accredited schools and programs of public health prior to the 2016 Council on Education for Public Health (CEPH) accreditation changes was unknown to the authors, but the CEPH requirement prior to 2016 was that all accredited schools and programs of public health offer the 5 core public health disciplines in their core curriculum.

c Discrete courses in 5 former core areas, introduction to public health in some cases, no other courses required for core (little to no change).

d Three to 5 discrete courses in former core areas, plus at least 1 additional course in or outside the former core area (some change).

e Two or fewer discrete courses in former core areas, disciplines outside former core areas, and/or courses combine and fuse multiple former core areas (extensive change).

Both schools and programs were less likely to change methods-focused courses than other courses in the core curriculum. A larger proportion of programs maintained the traditional biostatistics and epidemiology courses (88.5% and 90.8%, respectively); approximately two-thirds of schools maintained the traditional biostatistics and epidemiology courses (68.7% and 62.7%, respectively) (Table 5). Few schools and programs required a mixed-methods (quantitative and qualitative) course (10.7%) or stand-alone qualitative methods courses (8.1%).

**Table 5.** Types of courses in master of public health core curriculum at accredited schools and programs of public health, 2023^a^

CEPH competency domains	Course type	Schools of public health (n = 67)	Public health programs (n = 130)	Total (N = 197)
Evidence-based approaches to public health	Biostatistics^b^	68.7	88.5	81.7
	Epidemiology^b^	62.7	90.8	81.2
	Epidemiology and biostatistics combined	9.0	4.6	6.1
	Quantitative and qualitative/nonspecified methods	20.9	5.4	10.7
	Qualitative methods only	9.0	7.7	8.1
Public health and health care systems	Health policy and management^b^	44.8	40.8	42.1
	Health services/administration/management	38.8	54.6	49.2
Policy in public health	Policy and/or advocacy	13.4	30.8	24.9
	Social justice/equity	25.4	16.9	19.8
Planning and management to promote health	Social and behavioral health^b^	41.8	72.3	61.9
	Planning^c,d^	47.8	32.3	37.6
Leadership	Leadership	34.3	29.2	31.0
Communication	Communication	11.9	13.1	12.7
Not applicable	Ethics	13.4	16.2	15.2
Not applicable	Environmental health^b^	50.7	78.5	69.0

Abbreviation: CEPH, Council on Education for Public Health.

a All values are percentages.

b Traditional public health core discipline courses.

c Often combined with assessment, implementation, and/or evaluation.

d Includes only planning courses that are required in the absence of a traditional social and behavioral course. Programs with community health foci (20.0%) require a social and behavioral course and a planning course.

Half of all schools (50.7%) and more than three-quarters of all programs (78.5%) required MPH students to take an environmental health course. Across schools and programs combined, 69.0% required a discrete environmental health course.

Most schools (55.2%) and programs (59.2%) shifted from traditional health policy and management courses to offer courses that were focused on health services and administration and, to a lesser degree, policy. While 72.3% of programs continued to offer a traditional social and behavioral course, only 41.8% of schools continued to offer this course. Many schools of public health adopted planning courses (47.8%) or courses focused on health equity or social justice (25.4%) (Table 5).

## Discussion

The median number of courses in the MPH core, across schools and programs combined, decreased from 7 to 6 courses since reported in the 2020 MPH trends report.^8^ The fewest number of core courses in an MPH degree was 3, in a school, and the largest number of core courses was 15, in a single-concentration program.

The MPH core courses comprised a larger proportion of the overall curriculum for programs than for schools. While CEPH requirements do not specify the number of courses or credit hours for the MPH core curricula, generally, MPH concentration courses should comprise at least 20% (eg, 9 of 42 semester credits) for students to attain knowledge and skills in an area of specialization.^11^ In addition, the Blue Ribbon report recommended that no more than one-third of the MPH degree be composed of core courses.^1^ The larger proportion of core courses in programs was largely due to the fact that half of all programs had a single MPH concentration. Many single-concentration programs take a generalist approach to the MPH degree. According to the Blue Ribbon report, “Any public health curriculum must impart a broad range of skills and knowledge to help students understand how the world works.”^1^ Single-concentration programs are uniquely positioned to provide this breadth and address priority workforce needs in areas such as communication, respectful community engagement, systems thinking, teamwork, and health systems.^1,2,12^

The number of schools and programs adopting extensive changes to their MPH core curriculum increased by 153.6% from 2020 to 2023.^8^ The greater increase was among programs. However, nearly half of all schools made extensive core curriculum changes. Although most core curricula in programs were classified as category B (63.9%), with some core curricular change, this category decreased by 28.2% from 2020 to 2023.^8^

Schools and programs were more likely to make changes in health policy and management courses than in biostatistics and epidemiology courses. In an ongoing, associated qualitative study involving 18 academic affairs deans and program directors across 16 schools and programs, preliminary data show that educational leaders faced greater resistance to making changes to epidemiology and biostatistics courses (unpublished data, key informant interviews, conducted by C.L.P. and D.F.G.A.-N., February 10–September 1, 2023). With few schools requiring a mixed or qualitative methods course, we could not ascertain in which courses qualitative methods competencies were addressed. While more than three-quarters of programs require a discrete environmental health course, only half of all schools do. However, the combined total of schools and programs continuing to require a discrete environmental health course decreased by 6.8% from the 74.0% identified in the CEPH Environmental Health trends report in 2020.^13^ Few schools require their students to take an ethics course, although public health ethics could be interwoven into other courses.

### Limitations

This study had several limitations. First, an element of subjectivity existed in classifying courses into categories A, B, and C and mapping courses to CEPH foundational competency domains based on course titles. Titles of courses may not directly align with domains. We could not distinguish between core and required courses, so we recorded all required courses as core courses. This limitation was particularly relevant to single-concentration MPH degrees. Some courses were counted in more than 1 category based on their title (eg, leadership and administration). These findings represent public health school and program core curriculum data from April through July 2023, so additional changes may have occurred since that time.

## Conclusions

Most schools and programs of public health revised their MPH core curricula after accreditation changes in 2016 to better map to the foundational competencies. Future research will determine whether these curricular changes improve the knowledge and skill proficiency of public health graduates and the workforce.
